# Fitness costs of symbiont switching using entomopathogenic nematodes as a model

**DOI:** 10.1186/s12862-017-0939-6

**Published:** 2017-04-17

**Authors:** John G. McMullen, Brittany F. Peterson, Steven Forst, Heidi Goodrich Blair, S. Patricia Stock

**Affiliations:** 1 School of Animal and Comparative Biomedical Sciences, University of Arizona, 117 East Lowell Street, PO Box 210090, Tucson, AZ 85721 USA; 2 Current address: Department of Entomology, Cornell University, 2130 Comstock Hall, Ithaca, NY 14853 USA; 3 Center for Insect Science, University of Arizona, 1007 E. Lowell St, Tucson, AZ 85721 USA; 4 Department of Entomology, University of Arizona, 11490 E. South Campus Dr, Tucson, AZ 85721 USA; 5 Department of Biological Sciences, University of Wisconsin, Milwaukee, Lapham Hall 458, Milwaukee, WI 53201-0413 USA; 6 Current address: Department of Microbiology, University of Tennessee-Knoxville, F331A Walters Life Sciences, Knoxville, TN 37996-0845 USA

**Keywords:** Entomopathogenic nematodes, Symbiosis, Mutualism, Partner choice

## Abstract

**Background:**

Steinernematid nematodes form obligate symbioses with bacteria from the genus *Xenorhabdus*. Together *Steinernema* nematodes and their bacterial symbionts successfully infect, kill, utilize, and exit their insect hosts. During this process the nematodes and bacteria disassociate requiring them to re-associate before emerging from the host. This interaction can be complicated when two different nematodes co-infect an insect host.

**Results:**

Non-cognate nematode-bacteria pairings result in reductions for multiple measures of success, including total progeny production and virulence. Additionally, nematode infective juveniles carry fewer bacterial cells when colonized by a non-cognate symbiont. Finally, we show that *Steinernema* nematodes can distinguish heterospecific and some conspecific non-cognate symbionts in behavioral choice assays.

**Conclusions:**

*Steinernema-Xenorhabdus* symbioses are tightly governed by partner recognition and fidelity. Association with non-cognates resulted in decreased fitness, virulence, and bacterial carriage of the nematode-bacterial pairings. Entomopathogenic nematodes and their bacterial symbionts are a useful, tractable, and reliable model for testing hypotheses regarding the evolution, maintenance, persistence, and fate of mutualisms.

**Electronic supplementary material:**

The online version of this article (doi:10.1186/s12862-017-0939-6) contains supplementary material, which is available to authorized users.

## Background

Every plant and animal on this planet is engaged in beneficial and antagonistic relationships with microbes that profoundly influence their physiology, life history, and evolution. The ubiquity and biological importance of long-term, stable, and beneficial host-microbe associations, or mutualisms, have been the focus of many investigations [30, 33–36, 41, 56]. Models of mutualisms possess common factors that contribute to their success, maintenance, and perpetuation. Cooperative association, fitness benefits for all parties, partner fidelity and preference are key factors that influence the establishment and maintenance of mutualisms [12, 42].

In this respect, the relationship between Gram-negative *Xenorhabdus* bacteria and their *Steinernema* nematode hosts yields insights into processes that influence symbiont specificity, selection, and function. Furthermore, the inherent experimental tractability of these organisms and availability of genome sequences has spurred developments across a broad scientific front, such that this mutualism is now viewed as a model for basic research in ecology, evolution, biochemistry, and molecular genetics of symbiosis [4, 6, 8, 28, 33, 45–47].

Briefly, the *Xenorhabdus* life cycle depends on two eukaryote hosts, *Steinernema* nematodes and an insect (usually soil-dwelling, immature stages) for their survival, dissemination, and reproduction. The relationship between the bacteria and the nematodes is benign and mutually beneficial, whereas their relationship with the insect host is pathogenic [2, 16, 38]. After invading an insect, nematodes (third-stage infective juveniles) defecate *Xenorhabdus* symbionts into the hemocoel where they multiply rapidly, neutralize the insect’s immune system, induce septicemia and toxemia, and begin digesting the fat-rich insect cadaver. Within the compromised insect, nematodes mature, and sexually reproduce resulting in many thousands of offspring using the metabolic resources liberated from the cadaver [11, 55]. Once resources are exhausted, the nematodes develop into a non-feeding, environmentally-resistant “dauer” stage, known as the infective juvenile (IJ). Prior to emerging from the insect cadaver, IJs re-associate with *Xenorhabdus* symbionts, which colonize the IJ nematodes’ intestinal receptacle. IJs carrying bacterial symbionts persist in the soil until a new insect host can be infected. Nutrients provisioned by the nematode IJ are thought to support outgrowth and bacterial survival until the next insect host [29].

Each *Steinernema* species associates with a single *Xenorhabdus* species. However, one *Xenorhabdus* species can form associations with more than one *Steinernema* host [2, 47]. The most promiscuous *Xenorhabdus* species is *Xenorhabdus bovienii,* which associates with numerous *Steinernema* species comprising two different evolutionary clades in the *Steinernema* phylogeny [24, 25, 48, 49]. Previous studies revealed that the bacteria can contribute to virulence toward the insect host and support nematode reproduction [6, 9, 33, 44]. These benefits have also been demonstrated to have a strong phylogenetic correlation at the nematode intraspecific level [33] with the driving selective interest of nematode symbiont choice likely differing between nematode species [7].

Complicating matters, in nature, insects can become co-infected with two *Steinernema* species each harboring their respective bacterial symbiont [39]. This requires that nematodes disassociate with their bacterial partner, reproduce and mature within a mixed community, and then re-associate with the correct symbiont to avoid the fitness costs associated with harboring the wrong bacterium. This sets entomopathogenic nematode-bacterium symbioses apart from many other mutualistic systems where partners do not naturally disassociate under optimal environmental conditions.

Symbiont switching can have detrimental effects to animal hosts. In examples across invertebrate-microbe symbioses, symbionts serve important functions and provide adaptive features for their hosts by aiding in digestion, conferring pathogen/parasite resistance, and increasing host fitness [20, 23, 37, 43, 57]. In fact, within a single species of nematode, association with a closely-related non-cognate symbiont results in reduced fitness [33]. For this reason, partner fidelity and localization are especially important to host animals.

*Steinernema-Xenorhabdus* symbioses are excellent models for testing hypotheses concerning mutualism. Obligate mutualisms evolve and persist due to increased fitness of both parties through maintenance of partner fidelity and partner preference. Unlike many intracellular, vertically transmitted animal-microbe symbioses, entomopathogenic nematode-bacterial symbioses rely on partner re-association after each infection cycle. Due to the tractability of this system, we were able to test the fitness, fidelity, and preference of several nematode-bacterial partner pairs by switching their symbionts. We show that across a number of *X. bovienii*-*Steinernema* spp. combinations, cognate partners display the hallmarks of mutualism including increased fitness in cooperation, partner fidelity, and partner preference. Additionally, we show that the most derived *Steinernema* species tested *S. puntauvense*, is more promiscuous and is able to form relatively successful relationships with a wide variety of non-cognate *Xenorhabdus* species. Contrastingly, the ancestral *X. bovienii* host, *S. intermedium,* is the most fastidious of the nematode species examined, performing poorly with all non-cognate bacteria. *Xenorhabdus bovienii* (intermedium strain) is attractive to the largest variety of non-cognate nematode species. Taken together, these data show entomopathogenic nematode-bacterial mutualisms represent an important and useful model for understanding the evolution of mutualisms.

## Methods

### Nematode and Bacteria Isolates

Three *Steinernema* species (*S. intermedium, S. oregonense,* and *S. puntauvense*), which are hosts of *Xenorhabdus bovienii,* were used in this study to assess the cost/benefit of symbiont switching (Table 1). Two of these species (*S. oregonense* and *S. puntauvense*) were assessed in symbiont choice assays to determine symbiont preference (see more details below). Additionally, *Steinernema feltiae* and *Steinernema carpocapsae* were included (Table 1) in these assays given that these taxa have been extensively studied for the effects of symbiont switching events [7, 33, 45]. Details on nematode hosts and bacterial symbionts species/strain names, geographic origin, and sequence accession numbers are listed in Table 1. Identity of nematodes and bacteria considered in this study was molecularly confirmed prior to the initiation of the experiments by sequencing of 28S and 12S ribosomal DNA (rDNA) genes for the nematode hosts and 16S genes for their bacterial symbionts.

**Table 1:** *Steinernema* and *Xenorhabdus* species and strains considered in this study

*Steinernema* host species	Strain name and geographic origin	GenBank accession no. (12S/28S rRNA)	*Xenorhabdus* symbiont species	Bacterial strain abbreviation	GenBank accession no. 16S rRNA	Bacterial genome accession no.	Source
*S. carpocapsae*	All; USA	AY944007/ AF331900	*X. nematophila*	Xn	GU480972	FN887742	P. Stock
*S. feltiae*	FL; FL, USA	GU569030/ GU569049	*X. bovienii*	XbfFL	KF437819	PRJEB4320	K. Nguyen
*S. feltiae*	Moldova; Moldova	KF437815/ KF437816	*X. bovienii*	XbfM	KF437821	PRJEB4321	B. Adams
*S. feltiae*	SN; France	GU569031/ GU569050	*X. bovienii*	XbfSN	GU480976	PRJEB4319	P. Stock
*S. intermedium*	Type; SC, USA	AY944014/ AF331909	*X. bovienii*	Xbi	KF437822	PRJEB4327	P. Stock
*S. kraussei*	Quebec; Canada	GU569034/ GU569053	*X. bovienii*	XbkCA	KF437825	PRJEB4324	G. Belair
*S. kraussei*	Nemasys-L; USA	KF437817/ KF437818	*X. bovienii*	XbkBU	KF437824	PRJEB4325	Becker-Underwood
*S. jollieti*	Monsanto; MO, USA	GU569032/ GU569051	*X. bovienii*	Xbj	KF437823	PRJEB4326	Monsanto
*S. oregonense*	OS-10; OR, USA	AY944021/ AF331891	*X. bovienii*	Xbo	KF437826	PRJEB4323	P. Stock
*S. puntauvense*	Li6; Costa Rica	GU569037/ GU569056	*X. bovienii*	Xbp	KF437827	PRJEB4322	L. Uribe

Nematodes were reared in vivo with last instar wax moth larvae, *Galleria mellonella* (Lepidoptera: Pyrallidae) (Timberline Fisheries, Marion, IL) following procedures described by Kaya and Stock [22]. Modified White traps were used to harvest emerging infective juvenile stages (IJs), which were stored in tissue culture flasks at a concentration of approximately 3000 IJs/ml in 250 mL tissue culture flasks (BD Falcon, Franklin Lakes, 26 NJ, USA).

Bacterial symbionts were extracted from each nematode host species/strain by sonication of naturally colonized IJs. Bacterial stocks were established by growing each bacterial species/strain to mid-log phase in Luria-Bertani (LB) broth supplemented with 0.1% (*w*/*v*) sodium pyruvate [27, 58] grown in a shaking, dark incubator at 28 °C following procedures described by Akhurst [1]. Bacterial stocks were stored at −80 °C in LB broth supplemented with 20% (*v*/v) glycerol.

### Rearing of Aposymbiotic nematodes

Aposymbiotic (symbiont-free) nematodes were obtained via alkaline axenization of eggs harvested from first generation gravid females as described by McMullen and Stock [32]. Eggs were seeded on liver-kidney agar [47]. Aposymbiotic IJ nematodes were collected by removing the bottom portion with agar of the Petri dish and placing it in a sterile modified White trap [32]. IJs were stored in tissue culture flasks at 15 °C until use for experiments following the same procedures described above. The aposymbiotic nature of the IJs was confirmed by grinding them during the first and second week of emergence with a motorized pestle (Kontes™) in LB broth. The suspension was then plated onto differential nutrient agar supplemented with 0.0025% (*w*/*v*) bromothymol blue, 0.004% (*w*/*v*) triphenyl-tetrazolium, and 0.1% (*w*/*v*) sodium pyruvate (NBTA) [1] to confirm presence/absence of bacterial symbionts.

### Host-Symbiont Interactions Assays

#### Host virulence

Aposymbiotic IJs were surface sterilized according to procedures described by Stock and Goodrich-Blair [47]. IJ suspension as diluted in Grace’s medium to a concentration of 50 IJs in 10 μl. Bacteria cultures were grown overnight and then sub-cultured to a 1:100 dilution into LB broth and subsequently grown to OD_600nm_ (optical density at 600 nm) ~0.80. Bacterial suspension was then diluted in Grace’s insect medium (Sigma, St. Louis, MO) to a concentration 200 CFU (colony forming units) in 10 Μl*. prior* to injections, bacterial suspensions were plated to confirm that equal counts were used for all bacterial strains. The experimental arena was a 12-well plate. Each *G. mellonella* larvae was co-injected with a nematode/bacterium treatment. Treatments consisted of co-injections of different *Steinernema/Xenorhabdus* species/strain pairs (Table 2). Bacteria and nematodes were co-injected at the concentrations described above. Twelve *G. mellonella* larvae were used per treatment and each treatment were repeated three times independently. Insect mortality was monitored daily for 7 days post-injection. *G. mellonella* larvae were considered dead when they did not respond to the gentle prodding of soft-grip forceps. Resulting cadavers and progeny were used for all subsequent experiments, except for the choice assays.

**Table 2:**
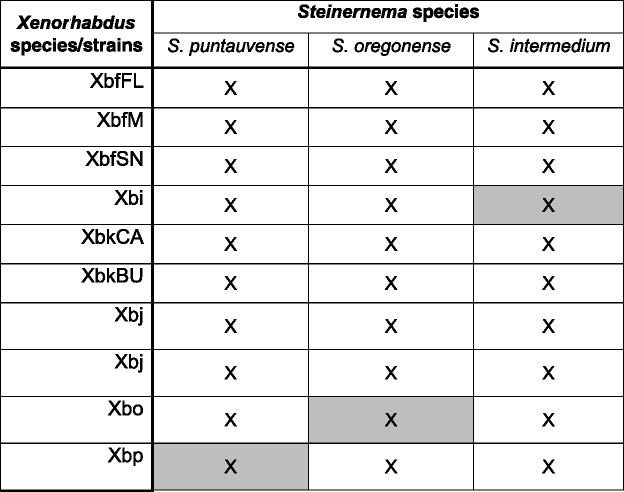
Cognate and non-cognate nematode-symbiont pairs considered in this study

Grey boxes denote cognate symbiont-nematode pair

#### Host reproductive fitness

Reproductive fitness of *S. intermedium*, *S. oregonense* and *S. puntauvense* was measured considering two parameters: a) infection productivity (i.e. percentage of cadavers producing progeny), and b) emerging IJ progeny (total number of emerging IJs).

For productive infection percentages, *G. mellonella* cadavers obtained from the virulence assays were considered. Each cadaver was thoroughly rinsed and transferred into a modified White trap at day seven post-injection. Cadavers were checked daily to record progeny emergence for up to 30 days. Cadavers producing at least 100 IJs were scored as ‘productive’, and productive infection percentage was calculated as the number of cadavers producing progeny out of the total number.

The emerging IJ progeny was determined by collecting the nematode suspension from each modified White trap daily for 10 days of post-emergence and following volumetric dilution procedures per Kaya and Stock [22].

#### Symbiont Carriage

IJ symbiont carriage was determined following procedures described by Goestch et al*.* [14]. Two technical replicates and three biological replicates were performed for each nematode-bacterium combination and averaged to obtain CFU IJ^−1^.

#### F1 Virulence

Virulence of the emerging IJ progeny (F1) was tested considering a one-on-one assay as described by Kaya and Stock [22]. The experimental arena was a 12-well plate, where each well was filled with 5 g of sterile sand. An inoculum of 50 IJs delivered in 100 μl of distilled water was poured onto the sand. One *G. mellonella* larvae was added into each well. The lid was placed and the plate was incubated at 20 °C in the dark. Mortality was monitored every 8 h for a total of 96 h. Experiments were repeated three times. For data analysis, percent mortality registered at 48 h was used, as it showed the largest difference between combinations.

#### Longevity of F1 Progeny

IJs obtained from the host reproductive fitness assays were examined 6-months post-emergence at 30× magnification. Nematodes were considered dead when they adopted a straight shape and/or showed no movement, whereas nematodes were considered live when they were moving or had a J-shape position. IJs were confirmed as dead by gentle prodding. Two technical replicates of at least 100 IJs were counted from each productive infection.

#### Morphometric analysis

The effect of cognate/non-cognate bacterial symbiont on nematode size was assessed by measuring length and width of IJ progeny (F1) obtained from each treatment. A total of 25 IJs/ treatment were randomly pooled. IJs were heat killed in M9 buffer at 50-60 °C [22] and subsequently fixed with triethanolamine formalin (TAF) following procedures described by Goodey [15]. IJs were mounted onto glass slides and measured using an Olympus BX51 microscope equipped with differential interference contrast optics and Olympus Microsuite software (Soft Imaging System Corp. CA, USA).

#### Bacteria choice assays

Choice assays were performed to test whether different *Steinernema* hosts of *X. bovienii* could discern between bacterial strains that are cognate vs. non-cognate. For this purpose, three *Steinernema* hosts of *X. bovienii* were considered: *S. feltiae* (SN strain), *S. oregonense* (OS-10 strain), and *S. puntauvense* (Li6 strain). In addition, two other *X. bovienii* strains were included, XbfFL (symbiont of S. feltiae strain FL) and Xbi (symbiont of *S. intermedium* Type strain) to account for symbionts that have a range of fitness effects on nematode hosts. *S. carpocapsae* (All strain) and its respective symbiont *X. nematophila* were considered for outgroup comparisons. For each nematode species used, a no-choice assay was performed where all bacterial foci in our test arena (Additional file 1: Fig. S1) consisted of the symbiotic partner as a control.

Aposymbiotic IJs were reared following procedures described by McMullen and Stock [32]. Prior to the experimental setup, nematodes were surface sterilized using the vacuum filtration method described by Kaya and Stock [22]. Due to low fitness of aposymbiotic *S. intermedium* nematodes, bacterial preference assays did not include this species as this would likely confound results.

Bacterial strains were grown from glycerol stocks overnight (12-16 h) in 5 ml of LB broth with 0.1% sodium pyruvate in a 15 ml centrifuge tube at 28 ± 1 °C in the dark with agitation. 50 μl of bacterial suspension was sub-cultured following the same conditions, but allowed to grow for 9 h prior to assay setup. Cultures were streaked onto NBTA to confirm liquid cultures were phase I under the same conditions described above.

Briefly, a 10 cm Petri dish was filled with 20 ml of lipid agar [1, 54] supplemented with 0.1% sodium pyruvate [58]. A bacterium inoculum of 10 μl was inoculated onto the agar plate in four equidistant positions, approximately 0.5 cm away from the edge. For each plate, one cognate and one non-cognate bacterial strain were applied so that two inoculum positions of the same strain were not next to each other (Additional file 1: Fig. S1). A 50 μl inoculum of 100 IJs was dispensed into the center of the plate. There were five replicates for each nematode-bacteria combination. Controls consisted of four foci of cognate bacteria. Plates were incubated at 20-25 °C for 3 days in the dark. This time period allowed for enough time for IJs to migrate to the preferred bacteria. Experiments were repeated twice, independently.

After 3 days, nematode migration to bacteria foci was evaluated. To facilitate visualization and counting of nematodes, bacteria foci and nematode inoculum area (center of plate) were cut and placed into a 5 cm Petri dish containing approximately 10 ml of M9 buffer. Dishes were then placed on a shaker overnight to expedite release of the nematodes embedded in the agar and bacterial lawn. The entire plate was then rinsed off to collect the IJs and stored in 50 ml centrifuge tubes at 4 °C until counted.

An attraction activity index (AAI) was calculated to assess nematode attraction for bacteria in the choice assays. For each treatment, the number of nematodes found in each bacterial focus (i.e. cognate or non-cognate) was combined. Similar to Zhang et al. [59], the formula below was used to obtain the AAI values. AAI ranges from 1 to −1 and the resulting values can be inferred as: 1 = all nematodes are attracted to non-cognate symbiont, 0 = equally prefer both bacteria, −1 = all nematode are repulsed by non-cognate strain. The formula used was: AAI = (number of nematodes in non-cognate bacterium) – (number of nematodes in cognate bacterium) / total number of nematodes in bacteria. For no-choice assay controls, bacterial foci pairs were randomly assigned to follow data analysis procedure used for choice assays.

#### Phylogenetic analyses

Phylogenetic analyses were conducted to determine relative phylogenetic distance between cognate and non-cognate *Xenorhabdus* strains. MEGA v.6 was used to generate both nematode and bacterial phylogenies [50]. For the *Xenorhabdus* phylogeny, the following sequences were aligned using MUSCLE [10] and concatenated: 16S rDNA, *recA, dnaN, gltX, gyrB,* and *infB* (Table 1, Additional file 1: Table S1). Maximum likelihood was carried out using a TN93 + G + I model of evolution with 1000 bootstrap replicates. The nematode phylogeny was generated using similar methods as the bacterial phylogeny with the mitochondrial and nuclear rRNA genes: 12S and 28S, respectively (Table 1). However, a GTR + G model of evolution was used. *S. carpocapsae* and its native symbiont *X. nematophila* were considered in nematode and bacterial phylogenies, respectively, as out group taxa.

#### Statistical Analyses

All statistical analyses were carried out using R, version 3.1.2 [40].

Nematode-bacterium virulence against *G. mellonella* were analyzed using a Cox mixed effects model (α = 0.05) from the package ‘coxme’ [52]. Each nematode host tested was analyzed separately and their cognate symbiont was used as the reference strain to build the Cox model. The bacterial strain was considered as a fixed effect in the model, while a random effect was included to better explain the variance due to differences between experimental setups. A *post hoc* Tukey HSD test was conducted using the ‘multcomp’ package [18]. Final insect mortality at day seven and median mortality time (LT_50_) were calculated from data assessed with the ‘survival’ package [51]. If a nematode-bacterium combination resulted in no deaths during the study period, a single death was added at day seven to allow the model to be stable.

Either a one-way analysis of variance (ANOVA) or mixed effects ANOVA (α = 0.05) was considered to analyze most assays. A one-way ANOVA was considered to analyze productive infection and F1 progeny data. In both instances, a single percentage was generated for each nematode-bacterium pair from each experimental setup. The remaining measures (progeny production, progeny survival, and symbiont carriage) were assessed with a mixed effect model. For all analyses, each nematode host was analyzed separately and the bacterial treatment was used as a fixed effect. When a mixed effect ANOVA was used, the experimental setup was used as a random effect to account for setup variation with the packages ‘lme4’ [3] and ‘car’ [13]. All residuals were visually inspected for normality and homoscedasticity. A *post hoc* Tukey HSD test was performed for pairwise comparisons between bacterial treatments with the package ‘lsmeans’ [26].

Nematode choice data was analyzed by performing a two-sided one-sample t-test for each AAI index with a μ = 0 (α = 0.05). *P*-values were adjusted using the Benjamini-Hochberg method to correct for false discovery rate in each nematode dataset.

Linear regression analyses (α = 0.05) were conducted on nematode and bacterial fitness and morphometric data. Nematode fitness was calculated as a factor of progeny count, progeny survival, and productive infection percentage relative to the cognate symbiont mean, while bacterial fitness was determined as a factor of nematode fitness and CFU IJ^−1^ relative to the average cognate symbiont mean. For morphometric analyses, the factor of length and width was analyzed. Each nematode host was analyzed separately.

## Results

All parameters measured across nematode-symbiont combinations were influenced by the pairing at hand with the exception of nematode size (width and length; Additional file 1: Fig. S2). Together the measured variables demonstrate the truly mutualistic nature of associations between *Steinernema* nematodes and their *Xenorhabdus* symbionts.

*Some non-cognate symbionts are sufficient to maintain virulence against insect hosts.*

All *Steinernema* spp. tested demonstrated greater virulence with their cognate symbionts than with any other, non-cognate bacterium. Of them, symbiont switching in *S. intermedium*, a clade I species, had the most pronounced impact on virulence. Association with the cognate symbiont, Xbi, with *S. intermedium* resulted in 50% insect mortality in 4 days and ultimately killed 100% of insect hosts within a week (Table 3). *S. intermedium* was also capable of moderate levels of virulence with both XbkBU and Xbj resulting in 58.3% and 66.7% mortality by day seven, respectively (Table 3). Association with XbfFL and Xbp did result in some insect mortality after 1 week, although LT_50_ was not observed within the 7-day observation period, and virulence when *S. intermedium* was colonized by these species was not statistically distinguishable from aposymbiotic *S. intermedium* or those species that were completely incompetent (Table 3).

**Table 3:** Non-cognate *X. bovienii* effect on nematode virulence

*X. bovienii* strains	*S. intermedium* (*n* = 36)^a^			*S. oregonense* (*n* = 36)^a^			*S. puntauvense* (*n* = 36)^a^		
	% Mortality^b^	LT_50_ (d)^c^	Rank^d^	% Mortality^b^	LT_50_ (d)^c^	Rank^d^	% Mortality^b^	LT_50_ (d)^c^	Rank^d^
None	0.0 ± 0	> 7	C	27.8 ± 0.07	> 7	BC	66.7 ± 0.08	4	BC
XbfFL	11.1 ± 0.05	> 7	C	22.2 ± 0.07	> 7	C	55.6 ± 0.08	3	AC
XbfM	0.0 ± 0	> 7	C	61.1 ± 0.08	4	B	80.6 ± 0.07	2	AB
XbfSN	0.0 ± 0	> 7	C	36.1 ± 0.08	> 7	BC	52.8 ± 0.08	4	C
Xbi	**100.0 ± 0**	**4**	**A**	50.0 ± 0.08	4	BC	77.8 ± 0.07	3	AC
Xbj	66.7 ± 0.08	4	AB	33.3 ± 0.08	> 7	BC	41.7 ± 0.08	> 7	AC
XbkBU	58.3 ± 0.08	4.5	B	41.7 ± 0.08	>7	BC	80.6 ± 0.07	2	A
XbkCA	0.0 ± 0	> 7	C	50.0 ± 0.08	7	BC	75.0 ± 0.07	3	AC
Xbo	0.0 ± 0	> 7	C	**91.7 ± 0.05**	**2**	**A**	58.3 ± 0.08	2	AC
Xbp	5.6 ± 0.04	> 7	C	50.0 ± 0.08	4	BC	**94.4 ± 0.04**	**2**	**A**

^a^Cognate symbiont data are bolded

^b^Percent mortality after 1 week with standard error

^c^Lethal time to kill 50% of population reported as days

^d^Letter-ranking generated from a Cox mixed effects model and post hoc Tukey test; see Additional file 1: Table S2 for summary of model

Contrastingly, both assayed clade III nematode species, *S. puntauvense* and *S. oregonense*, maintained some level of virulence with all tested bacteria and even without a bacterial partner (Table 3). As with *S. intermedium*, *S. oregonense* was most virulent when associated with their cognate symbiont; LT_50_ was shorter and 7-day insect mortality is higher in associations with Xbo (Table 3). *S. oregonense* and *S. puntauvense* exhibited moderate virulence with all *X. bovienii* tested and with no associated symbiont (aposymbiotic; Table 3). Interestingly, *S. puntauvense* had comparable LT_50_ times with many symbionts, though its cognate still produced the highest insect mortality at 7-days post-inoculation (Table 3).

### Symbiont switching has negative impacts on fitness

**Fig. 1:**
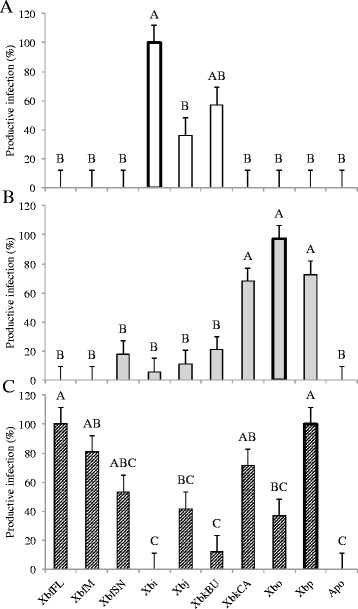
Productive infections resulting from *Steinernema-Xenorhabdus* experimental associations. Bars indicate the mean and SE resulting from a one-way ANOVA. Letters above bars show statistical ranking from *post hoc* Tukey test. *n* = 3 for each nematode-bacterium treatment. These values were calculated as the percent of cadavers that produce progeny in each independent experimental setup. Cognate symbionts are highlighted with a heavy outline. A. *S. intermedium*: F_9, 20_ = 8.3957, *p* < 0.0001, *R* ^2^ = 0.7907; B. *S. oregonense*: F_9, 20_ = 15.764, *p* < 0.0001, *R* ^2^ = 0.8764; and C. *S. puntauvense*: F_9, 20_ = 11.585, *p* 0.0001, *R* ^2^ = 0.8391

**Fig. 2:**
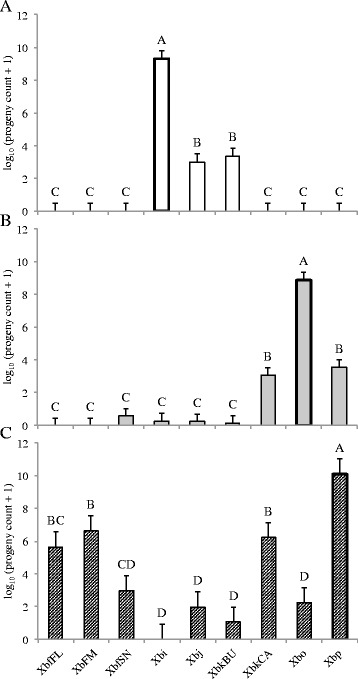
*Steinernema* progeny production with novel symbionts. Bars show the mean and SE resulting from a mixed effects ANOVA. Letters above bars demonstrate results from *post hoc* Tukey test. *n* = 36 for each nematode-bacterium treatment. Cognate symbionts are highlighted with a heavy outline. A. *S. intermedium*: F_8, 313_ = 91.015, *p* < 0.0001, *R* ^2^_O_ = 0.6917; B. *S. oregonense*: F_8, 313_ = 42.815, *p* < 0.0001, *R* ^2^_O_ = 0.5088; and C. *S. puntauvense*: F_8, 313_ = 22.398, *p* < 0.0001. *R* ^2^_O_ = 0.3478

Despite many *Steinernema-Xenorhabdus* combinations producing successful infections in insect hosts, associations with non-cognate symbionts impacted nematode reproductive success. The first measure of this impact was reflected in the proportion of productive infections from a given nematode-bacterium association. We determined an infection to be productive if it resulted in the emergence of at least 100 IJs. All species suffered negligible infection productivity when reared aposymbiotically and non-cognate associations were not sufficient to recover that loss in *S. intermedium* and *S. oregonense* (Fig. 1). *S. intermedium* had the least productive infections with non-cognate bacteria, with only XbkBU partially restoring infection productivity, and all combinations were not significantly different from the uncolonized aposymbiotic worms (including XbkBU; Fig. 1a). *S. intermedium* produced small numbers of IJs when partnered with Xbj and XbkBU (Fig. 2a). Associations between non-cognates XbkCA and Xbp generated a comparable proportion of productive infections to the cognate symbiont in *S. oregonense*; however, the total number of progeny from these infections was significantly less than what was measured from Xbo associations (Figs. 1b and 2b). In contrast, no progeny was recovered when *S. puntauvense* associated with non-cognate bacterium, even though most combinations resulted in productive infections (Figs. 1c and 2c). However, this pattern was not seen in combinations with Xbi, Xbj, XbkBU, nor Xbo (Fig. 1c).

**Fig. 3:**
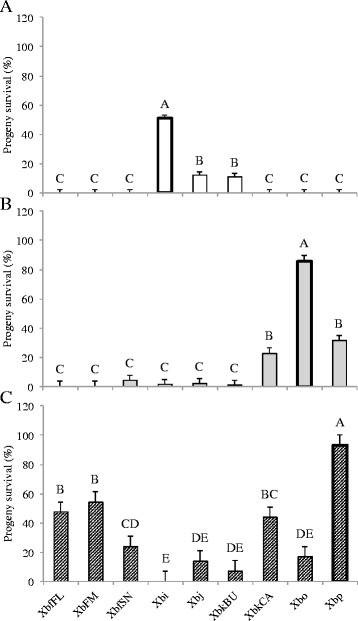
*Steinernema* progeny survival with non-cognate symbionts. Bars indicate the mean and SE resulting from a mixed effects ANOVA. Letters above bars show results from post hoc Tukey test. *n* = 36 for each nematode-bacterium treatment. Data represents percentage of nematode alive in a pool of 100 IJs. Cognate symbionts are highlighted with a heavy outline. A. *S. intermedium*: F_8, 313_ = 125.49, *p* < 0.0001, *R* ^2^_O_ = 0.7562; B. *S. oregonense*: F_8, 313_ = 57.75, *p* < 0.0001, *R* ^2^_O_ = 0.5846; and C. *S. puntauvense*: F_8, 313_ = 30.321, *p* < 0.0001, *R* ^2^_O_ = 0.4222

We also tested the longevity and virulence of the F1 IJs that emerged following association with non-cognate bacteria. In general, the patterns observed in IJ longevity followed the trends seen in progeny production. Nematode-symbiont pairings that yielded higher progeny production, also tended to have increased longevity. Six-months post-emergence, *S. intermedium-*Xbj and -XbkBU pairings had ~20% IJ survival, whereas *S. oregonense-*XbkCA and -Xbp pairings exhibited ~25% IJ survival (Fig. 3a & b). While these are both significantly less than the cognate symbiont, these pairings had higher survival than the other non-cognates that were capable of producing progeny (Fig. 3a & b). All symbiont pairings with *S. puntauvense,* except Xbi, had viable IJs 6-months post-emergence (~15-50%), though none of the non-cognate pairings survived as well as those associated with the cognate symbiont (Fig. 3c).

**Fig. 4:**
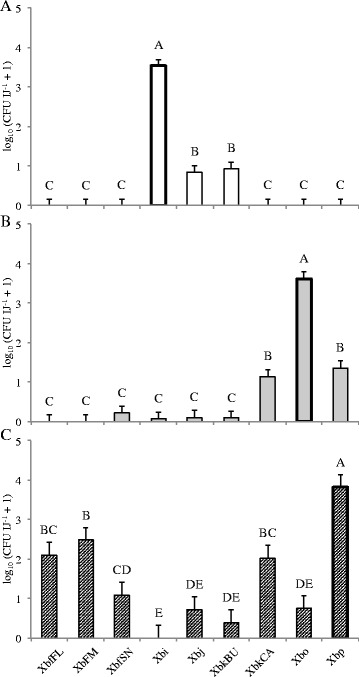
F1 progeny virulence against *G. mellonella.* Bars indicate the mean and SE resulting from a one-way ANOVA. Letters above bars show results from post hoc Tukey test. *n* = 3 for each nematode-bacterium treatment. Values were calculated as the percent of insects that died by 48 h in each independent experimental setup. Cognate symbionts are highlighted with a heavy outline. A. *S. intermedium*: F_8, 18_ = 6.857, *p* = 0.0004, *R* ^2^ = 0.7529; B. *S. oregonense*: F_8, 18_ = 20.846, *p* < 0.0001, *R* ^2^ = 0.9026; and C. *S. puntauvense*: F_8, 18_ = 34.312, *p* 0.0001, *R* ^2^ = 0.9385. post-

In addition to F1 IJ longevity, virulence of this new generation is imperative for nematode success long term and establishment of novel associations. Strikingly, F1 IJs from all non-cognate associations had significantly less virulence than cognate associations (Fig. 4). In combinations where progeny were produced some virulence was observed no matter how slight (Fig. 4). Consistent with other observations, *S. intermedium* was most susceptible to symbiont switching and resulted in a maximum of ~30% virulence in F1 IJs; whereas F1 *S. puntauvense* IJs had the most success with non-cognate symbionts and resulted in several combinations causing insect mortality >40% (Fig. 4a and c). *S. oregonense* had intermediate success out of the three nematodes tested, with an observed insect mortality below 40% (Fig. 4b).

### Non-cognate bacteria are less successful at colonizing IJs

**Fig. 5:**
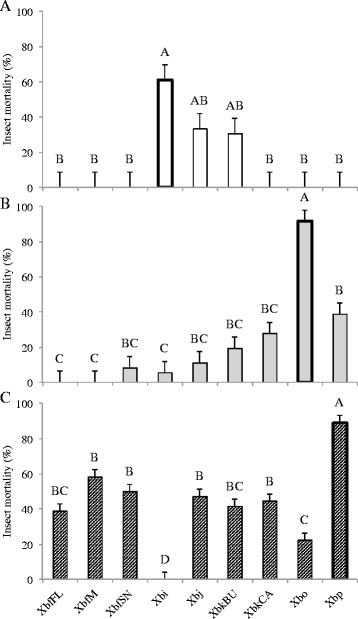
*Steinernema* symbiont carriage in F1 populations. Bars indicate the mean and SE resulting from a mixed effects ANOVA. Letters above bars show results from post hoc Tukey test. *n* = 36 for each nematode-bacterium treatment. Cognate symbionts are highlighted with a heavy outline. A. *S. intermedium*: F_8, 311_ = 138.72, *p* < 0.0001, *R* ^2^_O_ = 0.7755; B. *S. oregonense*: F_8, 313_ = 46.883, *p* < 0.0001, *R* ^2^_O_ = 0.5322; and C. *S. puntauvense*: F_8, 313_ = 24.467, *p* < 0.0001, *R* ^2^_O_ = 0.3780

We also assayed the extent to which bacteria were able to establish themselves in cognate and non-cognate IJs by measuring bacterial carriage. By and large, bacterial carriage follows the same patterns we saw in the other metrics discussed above. *S. intermedium* carried the fewest non-cognate bacteria (Fig. 5a)*, S. oregonense* can carry more species of non-cognate bacteria but at significantly fewer numbers (Fig. 5b), and *S. puntauvense* carried more non-cognate bacteria than the other two species though at lower concentrations than their cognate Xbp (Fig. 5c).

### *X. bovienii* hosts discern between some non-cognates in bacterial choice assays

**Fig. 6:**
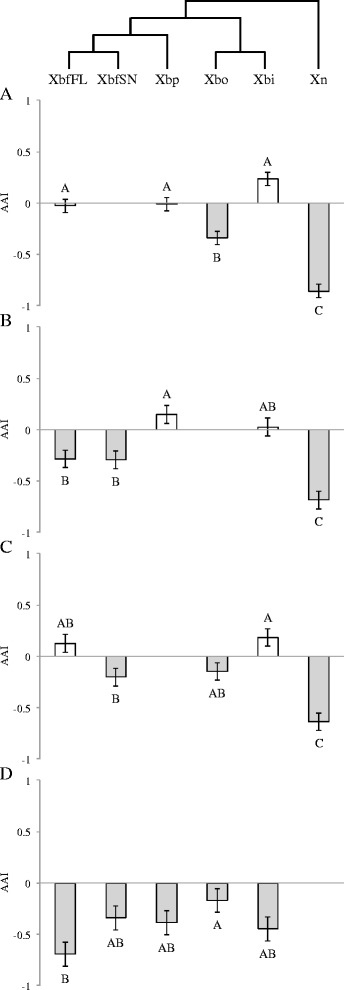
Nematode preference for non-cognate symbionts. A. *S. feltiae,* B. *S. oregonense,* C. *S. puntauvense,* and D. *S. carpocapsae.* Bars represent the mean and SE resulting from two-sided one-sample t-test analyses with μ = 0. *P*-values were adjusted with Benjamini-Hochberg method to correct for the false discovery rate and their results are indicated by the following symbols: NS = non-significant, * = *p* < 0.05, ** = *p* < 0.01, *** = *p* < 0.001, **** = *p* < 0.0001. *n* = 10 for each AAI (attraction activity index) value. *White bars* indicate nematode attraction to non-cognate symbiont, while gray bars show repulsion. Cognate symbiont bars are highlighted with a heavy outline. See Additional file 1: Table S3 for summary statistics

Using choice assays, we determined that *Steinernema* IJs were able to discern between some conspecific non-cognate bacterial strains and are repelled by heterospecific bacteria (Fig. 6). For example, *S. carpocapsae* was not attracted to any of the *X. bovienii* strains (mean AAI range: −0.3 to −0.7), with the exception of Xbo (AAI = 0.2). The nematodes appeared to not be able to distinguish between this bacterium (Xbo) and their cognate partner (Fig. 6d). Similarly, natural *X. bovienii* hosts (*S. feltiae*, *S. oregonense*, and *S. puntauvense*) were not attracted to Xn (mean AAI range of −0.6 to −0.9) (Fig. 6a-c). *Steinernema* species that naturally associate with *X. bovienii* exhibited strain-specific behavioral preferences within *X. bovienii* (Fig. 6a-c). For instance, *S. feltiae* preferred XbfFL and Xbp equally as it did for its cognate symbiont. This nematode was also attracted to Xbi but repelled Xbo (Fig. 6a). *S. oregonense* was not attracted to XbfFL and XbfSN, and did not demonstrate a differentiated preference for Xbp and Xbi and its cognate (Fig. 6b). *S. puntauvense* did not display a preference for any of the tested conspecific bacteria (Fig. 6c). Interestingly, Xbi was the only non-cognate bacterium that was always preferred (mean AAI range: 0.02-0.2) by the three *X. bovienii* nematode host species tested in this study (Fig. 6a-c).

### Phylogeny as a predictor of fitness effects of non-cognate associations

**Fig. 7:**
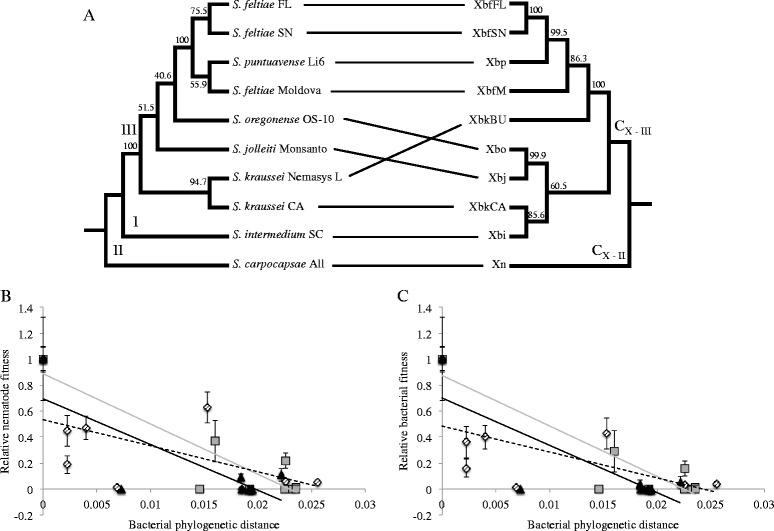
Evolutionary relatedness between *Steinernema* nematodes and their native symbionts and the fitness consequence of host-symbiont switching events. A. Co-phylogeny tanglegram of *Steinernema-Xenorhabdus* pairs used in experiments. The *Steinernema* phylogeny was generated with concatenated 12S and 28S rRNA genes (see Table 1 for accession IDs), while the *Xenorhabdus* phylogeny shows the results from a concatenation of the following genes: 16S rRNA, *recA*, *dnaN*, *gltX*, *gyrB*, and *infB* (see Additional file 1: Table S1 for gene labels and Table 1 for genome accession IDs). Internal nodes indicate bootstrap replicate values >50%. Panels B and C show relative nematode and bacterial fitness, respectively, by bacterial phylogenetic distance from native *Steinernema-Xenorhabdus* pairing. For both graphs, data points indicate mean and SE from a linear regression analysis; see Additional file 1: Table S4 for model summary. *S. intermedium* = *black triangles* with *black line*, *S. oregonense* = *gray squares* with *gray line*, and *S. puntauvense* = hashed diamonds with *dashed line*

A co-phylogenetic hypothesis for all bacteria-nematode pairs considered in this study was developed (Fig. 7a). The overall fitness effects of all nematode-bacterial pairings were regressed against the phylogenetic distance between the test bacterium and the cognate bacterium (Fig. 7b & c). In all cases, we show that fitness of both bacteria and nematodes declines as the phylogenetic distance between the cognate and non-cognate bacteria increases (Fig. 7b & c). These results suggest that the more distant a bacterial strain is in relation to a nematode’s cognate symbiont, the more detrimental the fitness effects are on non-cognate nematode partner. In particular, *S. intermedium* and *S. oregonense* are more sensitive to association with a phylogenetically distant symbiont, with 3.5-3.8 unit decrease in nematode and bacterial fitness for every 0.1 nucleotide change in symbiont (Additional file 1: Table S4). *S. puntauvense* is more tolerant to association with non-cognates and only suffers a 1.9-2.0 unit decrease in fitness for every 0.1 bacterial nucleotide change (Additional file 1: Table S4).

## Discussion

Entomopathogenic nematodes and their bacterial partners are an important model system for studying mutualism. The data presented here demonstrate that *Steinernema-Xenorhabdus* symbioses are mutually beneficial with regard to nematode and bacterial fitness across multiple species of nematode hosts. Association with cognate symbionts benefits the nematode host in terms of virulence against insect hosts, total progeny production, F1 progeny longevity, and virulence. The bacterial partners also benefit by increased host colonization, and thus have an increased chance of being vectored into a new insect host, by their nematode host. The final piece of this puzzle is partner fidelity. Not only did nematodes discern among different bacterial strains to varying degrees, but they tended to prefer bacteria which ultimately increase their virulence, longevity, and reproductive success.

Generally, all cognate nematode-bacteria pairs outperformed all other combinations in terms of virulence, progeny production and longevity, bacterial carriage, and fitness of both parties. With the exception of nematode size (length and width; Additional file 1: Fig. S2), all measured variables were influenced by symbiont partner associated with the given nematode host. Nematodes were also capable of discerning their cognate bacterium in a choice assay and tended to prefer their native symbiont to heterospecific strains, while their attraction toward non-cognate symbionts mirrored fitness and virulence trends measured in this study (Fig. 6). Most importantly, the impacts for both nematode and bacterial fitness were correlated to the phylogenetic distance between the cognate bacterium and that colonizing partner (Fig. 7b & c). These data solidify the argument that entomopathogenic nematodes and their bacterial symbionts represent a true mutualistic symbiosis.

While this trend is consistent across species, each tested nematode species had a distinct pattern of effects when associated with various *X. bovienii* strains. For example, *S. intermedium*, the only species member of clade I tested, was very fastidious. This nematode showed the highest virulence, progeny production, and F1 longevity with its native symbiont, Xbi (Table 3, Figs. 2a, 3a and 4a). However, these same metrics were only minimally restored when the nematodes associated with XbkBU and Xbj; no non-cognate symbiont restored even 50% virulence, IJ production, or progeny success as the native Xbi (Table 3, Figs. 2a, 3a and 4a). Unfortunately, due to technical limitations, we were not able to determine bacterial preference in *S. intermedium*, but we did assay how other *Steinernema* species responded to Xbi. Interestingly, Xbi was attractive to non-cognate nematode species such as *S. feltiae, S. oregonense* or *S. puntauvense,* despite conferring almost no fitness or virulence benefits (Table 3, Figs. 6a–c, & 7b). Given that its host, *S. intermedium* is in a basal (or more ancestral) phylogenetic clade to these other *X. bovienii* hosts tested, Xbi may exhibit some attractive trait which is generic to all *X. bovienii,* and as such is detectable by all *X. bovienii* host nematodes. In this respect, it could be speculated an attraction trait, present in *Xbi*, which more derived nematode hosts are cuing in on, may also be present in the cognate *X. bovienii* lineages. It has been shown that differences in primary and secondary metabolite biosynthetic capabilities, amino acid metabolic capabilities, and a number of uncharacterized genes detected in the genomes of *X. bovienii* strains maybe be discernable by host nematodes [33]. This was supported by the fact that *S. carpocapsae,* which does not carry *X. bovienii*, was not attracted to any *X. bovienii* bacteria assayed in this study, with the exception of Xbo (Fig. 6d).

*Steinernema oregonense* followed a similar pattern, as it performed best with its cognate symbiont in all metrics assessed. For example, we showed that *S. oregonense-*Xbp and -XbkCA combinations had moderate success in terms of progeny production, longevity, and bacterial carriage but failed to restore performance to the level of associations with the cognate symbiont (Figs. 2b, 3b and 4b). Additionally, *S. oregonense* was attracted to Xbp, although not significantly (Fig. 6b), but perhaps this reflects the nematode’s ability to detect some indication of its benefit over other less advantageous bacteria. It could then be hypothesized that there are phenotypes of bacterial symbionts that the nematode can discriminate and react to behaviorally, such as chemical cues or signals they can perceive.

This phenomenon has been observed in associations of many taxa where host/partner finding is an important part of the symbiosis. For example, leaf-cutting ants discriminate between concolonial, allocolonial, and allospecific fungi which differed measurably in the volatile, chemical signatures [53]. Beewolves recognize the colonization by opportunistic, non-symbiotic bacteria and do not pass those bacteria on to their offspring into brood cells [21]. In choice assays, both ambrosia beetles and hermit crabs using olfactory-based chemical discrimination to locate their symbiotic partners, fungi and sea anemones respectively, [5, 19]. Therefore, it is plausible that partner fidelity in entomopathogenic nematode-bacterium symbioses may be, in part, chemically mediated.

In contrast, our data showed that *S. puntauvense* was symbiotically promiscuous, demonstrating comparable infection success and progeny production with any *X. bovienii feltiae* bacterium (Xbf*) as it did with Xbp (its cognate symbiont). We also showed that *S. puntauvense* was attracted to two non-cognate symbionts, XbfSN and Xbi, in bacterial preference assays. The preference of XbfSN may reflect phylogenetic relatedness of Xbp to Xbf* bacteria, suggesting that this clade may share common features which facilitate success in the first generation of cross-association but cannot be maintained in subsequent generations. This was manifested in the observed limited virulence and longevity of F1 *S. puntauvense*-Xbf* combinations (Figs. 3c and 4c), and may be also due to insufficient bacterial carriage in the IJs (Fig. 5). These findings are supported by the attraction assays in *S. feltiae* (Fig. 6a) and other measures of fitness and virulence [33].

The demonstrated partner fidelity we see in these *Steinernema* species coupled with the resulting multi-generational benefits of cognate associations are important for symbiotic stasis. It is possible for an insect to become co-infected with more than one nematode species (carrying their respective symbionts) simultaneously. In doing so, nematodes must be able to identify their cognate symbiont or risk the fitness costs as manifested in the present study. To complicate things further, *X. bovienii* strains exhibit differential virulence within an insect host [31]. These observations suggest certain *X. bovienii* strains can directly outcompete other less virulent strains [31]. Additionally, it is possible that distinct strains of *X. bovienii* may have distinct nutritional requirements and/or differences in metabolic capability which could impact the nematode carrying them. Thus, the ability to discriminate native bacterial partners within a mixed infection is imperative for nematode success.

Alternatively, nematode discrimination may be a mechanism to prevent co-infection events thereby reducing within host competition between different *Xenorhabdus* strains. Previous evidence suggests that some Steinernematid IJs respond to volatile chemical cues from insect hosts infected by both conspecifics and heterospecifics, and this chemical profile is influenced by the *Xenorhabdus* symbionts [17]. However, previous phylogenetic data leads to the hypothesis that host-symbiont switching events are rampant throughout the *Steinernema-Xenorhabdus* co-phylogeny which would suggest the ability of IJs to discern *Xenorhabdus* partners that are best suited for nematode success.

*Steinernema-Xenorhabdus* symbioses exhibit all the hallmarks of mutualism increased fitness for both partners, divided but equally important roles in cooperative infections, and preference for native symbionts [12]. Additionally, the fitness costs of *Steinernema-Xenorhabdus* non-cognate associations are predictable based on phylogeny (Fig. 7).

## Conclusions

Entomopathogenic nematodes and their bacterial symbionts provide an ideal model system for studying mutualism because of their manipulability, short generation times, and ease of rearing. These stable associations carry measurable fitness benefits, have strong partner preference, and are phylogenetically predictable. We have demonstrated the effects of partner-switching manifest across three species of *Steinernema* nematodes which host different *X. bovienii* strains as symbionts. Each nematode species performed best in terms of reproductive fitness and virulence when associated with its cognate symbiont. IJs colonized with native partners harbor more bacteria per nematode thus increasing the likelihood of the bacterium being passaged to offspring in a new insect host successfully. Finally, these associations were reinforced by strong partner preference evidence, shown in the symbiont choice assays, meaning *X. bovienii* strains produce reliable cues or signals that *Steinernema* nematodes were able to detect and distinguish to varying degrees. Using the entomopathogenic nematode-bacterium association as a model for mutualism provides a practical avenue to study the ecological and evolutionary consequences of co-evolved symbioses.

## Additional file

Additional file 1:
**Figure S1.** Effect of non-cognate symbionts on nematode body size. **Fig. S2** Schematic representation of setup for preference assays. **Table S1** Bacterial gene labels of sequences used for bacterial phylogeny. **Table S2** Summary results from Cox mixed effects models. **Table S3** Summary statistics for choice assays. **Table S4** Summary results of linear regression models. (DOCX 61 kb)

## Abbreviations

AAI: attractions activity index
ANOVA: analysis of variance
CFU: colony forming unit
DNA: deoxyribonucleic acid
F1: first generation progeny
IJ: infective juvenile
LB: Luria-Bertani
LT_50_: median lethal time
NBTA: nutrient agar supplemented with 0.0025% (*w*/*v*) bromothymol blue, 0.004% (*w*/*v*) triphenyl-tetrazolium, and 0.1% (*w*/*v*) sodium pyruvate
OD: optical density
rDNA: ribosomal DNA
RNA: ribonucleic acid
rRNA: ribosomal RNA
TAF: triethanolamine formalin
Xbf*: any *X. bovienii* strain associated with any *S. feltiae* strain
XbfFL: *X. bovienii* associated with *S. feltiae* FL
XbfM: *X. bovienii* associated with *S. feltiae* Moldova
XbfSN: *X. bovienii* associated with *S. feltiae* SN
Xbi: *X. bovienii* associated with *S. intermedium* Type
Xbj: *X. bovienii* associated with *S. jollieti* Monsanto
XbkBU: *X. bovienii* associated with *S. kraussei* Nemasys-L
XbkCA: *X. bovienii* associated with *S. kraussei* Quebec
Xbo: *X. bovienii* associated with *S. oregonense* OS-10
Xbp: *X. bovienii* associated with *S. puntauvense* Li6
Xn: *X. nematophila* associated with *S. carpocapsae* All
