# Supplementary material for: Fitness costs of symbiont switching using entomopathogenic nematodes as a model
Source: BMC Evol Biol. 2017 Apr 17;17:100. doi: 10.1186/s12862-017-0939-6 (PMC5392933; doi:10.1186/s12862-017-0939-6)
Supplement: Additional file 1: — Figure S1. Effect of non-cognate symbionts on nematode body size. Fig. S2 Schematic representation of setup for preference assays. Table S1 Bacterial gene labels of sequences used for bacterial phylogeny. Table S2 Summary results from Cox mixed effects models. Table S3 Summary statistics for choice assays. Table S4 Summary results of linear regression models. (DOCX 61 kb) [file 12862_2017_939_MOESM1_ESM.docx]

**Additional files**

Figure S2. Schematic representation of setup for preference assays.

In this diagram, positions A-D indicate bacterial inoculation sites. A and C = cognate bacterium, B and D = non-cognate bacterium, and E = nematode inoculum site. All bacteria positions are equidistant from the nematodes inoculation position.

A.

B.

Figure S1. Effect of non-cognate symbionts on nematode body size

Points represent the mean and SE of nematode body size with respect to relative bacterial phylogenetic distance. See supplemental file 3 for linear regression model statistics. *S. intermedium* = black triangles with black line, *S. oregonense* = gray squares with gray line, and *S. puntauvense* = hashed diamonds with dashed line.

Table S1: Bacterial gene labels of sequences used for bacterial phylogeny

| *X. bovienii* strains | **Genes** | | | | | |
| --- | --- | --- | --- | --- | --- | --- |
|  | 16s rRNA | *dnaN* | *gltX* | *gyrB* | *infB* | *recA* |
| XbfFL | XBFFL1v2_16s_rRNA_1 | XBFFL1v2_2280026 | XBFFL1v2_2220025 | XBFFL1v2_2280028 | XBFFL1v2_310078 | XBFFL1v2_2510006 |
| XbfM | XBFM1v2_16s_rRNA_1 | XBFM1v2_1660008 | XBFM1v2_2260002 | XBFM1v2_1660010 | XBFM1v2_1260074 | XBFM1v2_520006 |
| XbfSN | XBFFR1v2_16s_rRNA_1 | XBFFR1v2_2180042 | XBFFR1v2_1670001 | XBFFR1v2_2180040 | XBFFR1v2_1840078 | XBFFR1v2_2220005 |
| Xbi | XBI1v2_16s_rRNA_1 | XBI1v2_280046 | XBI1v2_2040001 | XBI1v2_280048 | XBI1v2_1570072 | XBI1v2_2760021 |
| Xbj | XBJ2v2_16s_rRNA_1 | XBJ2v2_2360042 | XBJ2v2_1630001 | XBJ2v2_2360040 | XBJ2v2_130060 | XBJ2v2_1700008 |
| XbkBU | XBKB1v2_16s_rRNA_1 | XBKB1v2_2910007 | XBKB1v2_2200001 | XBKB1v2_2910009 | XBKB1v2_20054 | XBKB1v2_260006 |
| XbkCA | XBKQ1v2_16s_rRNA_1 | XBKQ1v2_2100064 | XBKQ1v2_1410002 | XBKQ1v2_2100066 | XBKQ1v2_760023 | XBKQ1v2_2380024 |
| Xbo | XBO1v2_16s_rRNA_1 | XBO1v2_2060041 | XBO1v2_2310004 | XBO1v2_2060039 | XBO1v2_1780013 | XBO1v2_2080013 |
| Xbp | XBP1v2_16s_rRNA_1 | XBP1v2_2070042 | XBP1v2_840001 | XBP1v2_2070040 | XBP1v2_2990056 | XBP1v2_2940022 |
| Xn | XNC1_rRNA0001 | XNC1_0002 | XNC1_3205 | XNC1_0004 | XNC1_0505 | XNC1_1261 |

Table S2: Summary results from Cox mixed effects models

| *S. intermedium* (integrated χ^2^ = 288.63, df = 10, p-value < 0.0001) | | | | |
| --- | --- | --- | --- | --- |
| *X. bovienii* strain | Coefficient (± SE) | Hazard ratio | z-score | p-value |
| Xbi (reference) |  |  |  |  |
| None | -5.36 ± 1.03 | 0.004 | -5.20 | 2.0e-07 |
| XbfFL | -3.94 ± 0.56 | 0.019 | -7.05 | 1.8e-12 |
| XbfM | -5.36 ± 1.03 | 0.005 | -5.20 | 2.0e-07 |
| XbfSN | -5.35 ± 1.03 | 0.005 | -5.20 | 2.0e-07 |
| Xbj | -0.82 ± 0.27 | 0.439 | -3.00 | 0.0027 |
| XbkBU | -1.31 ± 0.29 | 0.271 | -4.43 | 9.4e-06 |
| XbkCA | -5.36 ± 1.03 | 0.005 | -5.20 | 2.0e-07 |
| Xbo | -5.36 ± 1.03 | 0.005 | -5.20 | 2.0e-07 |
| Xbp | -4.63 ±0.75 | 0.010 | -6.16 | 7.1e-10 |
| *S. oregonense* (integrated χ^2^ = 72.37, df = 10, p-value < 0.0001) | | | | |
| *X. bovienii* strain | Coefficient (± SE) | Hazard ratio | z-score | p-value |
| Xbo (reference) |  |  |  |  |
| None | -2.08 ± 0.37 | 0.125 | -5.68 | 1.3e-08 |
| XbfFL | -2.33 ± 0.40 | 0.098 | -5.83 | 5.5e-09 |
| XbfM | -1.00 ± 0.28 | 0.368 | -3.59 | 3.3e-04 |
| XbfSN | -1.65 ± 0.33 | 0.191 | -4.97 | 6.7e-07 |
| Xbi | -1.22 ± 0.30 | 0.296 | -4.10 | 4.1e-05 |
| Xbj | -1.84 ± 0.34 | 0.159 | -5.38 | 7.4e-08 |
| XbkBU | -1.54 ± 0.32 | 0.214 | -4.88 | 1.0e-06 |
| XbkCA | -1.33 ± 0.30 | 0.263 | -4.48 | 7.3e-06 |
| Xbp | -1.31 ± 0.30 | 0.270 | -4.40 | 1.1e-05 |
| *S. puntauvense* (integrated χ^2^ = 52.07, df = 10, p-value < 0.0001) | | | | |
| *X. bovienii* strain | Coefficient (± SE) | Hazard ratio | z-score | p-value |
| Xbp (reference) |  |  |  |  |
| None | -0.95 ± 0.27 | 0.387 | -3.53 | 4.1e-04 |
| XbfFL | -0.85 ± 0.28 | 0.426 | -3.01 | 0.0026 |
| XbfM | -0.16 ± 0.25 | 0.848 | -0.65 | 0.52 |
| XbfSN | -1.16 ± 0.29 | 0.313 | -4.02 | 5.8e-05 |
| Xbi | -0.49 ± 0.26 | 0.612 | -1.92 | 0.055 |
| Xbj | -1.40 ± 0.31 | 0.247 | -4.48 | 7.4e-06 |
| XbkBU | 0.00 ± 0.25 | 0.999 | 0.00 | 1.0 |
| XbkCA | -0.60 ± 0.26 | 0.550 | -2.31 | 0.021 |
| Xbo | -0.74 ± 0.28 | 0.475 | -2.66 | 0.0077 |

Table S3: Summary statistics for choice assays

| **Treatment** | **t-statistic** | **df** | **Raw p-value** | **Adjusted p-value*** |
| --- | --- | --- | --- | --- |
| ***S. carpocapsae*** | | | | |
| Control (Xn) | -0.7182 | 9 | 0.4908 | 0.4908 |
| XbfFL | -6.8731 | 9 | < 0.0001 | *0.0004* |
| XbfSN | -3.7972 | 9 | 0.0042 | *0.0127* |
| Xbi | -3.2183 | 9 | 0.0105 | *0.0158* |
| Xbo | -1.2509 | 9 | 0.2425 | 0.291 |
| Xbp | -3.2947 | 9 | 0.0093 | *0.0158* |
| ***S. feltiae*** | | | | |
| Control (XbfSN) | -0.2501 | 9 | 0.8082 | 0.8670 |
| XbfFL | -0.3641 | 9 | 0.7242 | 0.8670 |
| Xbi | 3.9603 | 9 | 0.0033 | *0.0066* |
| Xbo | -3.978 | 9 | 0.0032 | *0.0066* |
| Xbp | -0.1723 | 9 | 0.867 | 0.867 |
| Xn | -20.6798 | 9 | < 0.0001 | *< 0.0001* |
| ***S. oregonense*** | | | | |
| Control (Xbo) | 0.5533 | 9 | 0.5936 | 0.7123 |
| XbfFL | -2.8701 | 9 | 0.0185 | *0.0369* |
| XbfSN | -3.8227 | 9 | 0.0041 | *0.0122* |
| Xbi | 0.2293 | 9 | 0.8237 | 0.8237 |
| Xbp | 1.9125 | 9 | 0.0881 | 0.1322 |
| Xn | -10.3063 | 9 | < 0.0001 | *< 0.0001* |
| ***S. puntauvense*** | | | | |
| Control (Xbp) | 0.8824 | 9 | 0.4005 | 0.4005 |
| XbfFL | 1.669 | 9 | 0.1295 | 0.1794 |
| XbfSN | -1.5757 | 9 | 0.1495 | 0.1794 |
| Xbi | 2.5902 | 9 | 0.0292 | 0.0876 |
| Xbo | -1.9136 | 9 | 0.0880 | 0.1759 |
| Xn | -9.6498 | 9 | < 0.0001 | *< 0.0001* |

*Raw p-values were adjusted using the Benjamini-Hochberg method. Significant p-values are italicized.

Table S4: Summary results of linear regression models

| **Nematode fitness** | | | | | |
| --- | --- | --- | --- | --- | --- |
| Nematode host | Coefficient (± SE) | t-value | df | p-value | R^2^ |
| *S. intermedium* | -35.23 ± 5.40 | -6.52 | 322 | 2.72e-10 | 0.1166 |
| *S. oregonense* | -38.77 ± 3.19 | -12.16 | 322 | < 2e-16 | 0.4153 |
| *S. puntauvense* | -20.13 ± 3.15 | -6.39 | 322 | 5.75e-10 | 0.1126 |
| **Bacterial fitness** | | | | | |
| Nematode host | Coefficient (± SE) | t-value | df | p-value | R^2^ |
| *S. intermedium* | -36.52 ± 5.19 | -7.037 | 320 | 1.20e-11 | 0.134 |
| *S. oregonense* | -38.84 ± 2.68 | -14.51 | 322 | < 2e-16 | 0.3953 |
| *S. puntauvense* | -19.88 ± 2.74 | -7.255 | 322 | 3.03e-12 | 0.1405 |
| **Nematode body size** | | | | | |
| Nematode host | Coefficient (± SE) | t-value | df | p-value | R^2^ |
| *S. intermedium*^*^ | -204393.0 ± 27686.8 | -7.382 | 298 | 1.56e-12 | 0.1546 |
| *S. oregonense*^**^ | 181823.3 ± 28921.3 | 6.287 | 698 | 5.71e-10 | 0.0522 |
| *S. puntauvense*^***^ | 48951.5 ± 21860.1 | 2.239 | 1098 | 0.0253 | 0.0045 |

* All nematode bacterium combinations included 100 replicates when possible (50 IJs measured from two of the three experimental setups since one only resulted in progeny with Xbi), except for: XbfFL (n = 0), XbfM (n = 0), XbfSN (n = 0), XbkCA (n = 0), Xbo (n = 0), Xbp (n = 0).

** All nematode bacterium combinations included 150 replicates when possible (50 IJs measured from each experimental setup), except for: XbfFL (n = 0), XbfM (n = 0), XbfSN (n = 50), Xbi (n = 50), Xbj (n = 50), XbkBU (n = 100).

*** All nematode bacterium combinations included 150 replicates when possible (50 IJs measured from each experimental setup), except for: Xbi (n = 0), XbkBU (n = 50).
